# Fatty Acids as Prebiotics and Their Role in Antibiofilm Activity

**DOI:** 10.3390/antibiotics15010057

**Published:** 2026-01-05

**Authors:** Filomena Nazzaro, Francesca Coppola, Florinda Fratianni, Raffaele Coppola

**Affiliations:** 1 Institute of Food Sciences, National Research Council (CNR), Via Roma 64, 83100 Avellino, Italy; florinda.fratianni@isa.cnr.it; 2 Department of Agricultural Sciences, University of Naples Federico II, Piazza Carlo di Borbone 1, 80055 Portici, Italy; francesca.coppola2@unina.it; 3 Department of Agricultural, Environmental and Food Sciences, University of Molise, Via De Sanctis, 86100 Campobasso, Italy; coppola@unimol.it

**Keywords:** fatty acids, prebiotics, antimicrobial resistance, biofilm, pathogens

## Abstract

Microbial biofilms pose significant medical and industrial challenges due to their resistance to conventional antimicrobials, accounting for 40–80% of bacteria in various environments. This resistance primarily results from the extracellular polymeric matrix, a protective network of sugars, proteins, and other molecules produced by bacteria. The matrix restricts antibiotic penetration, facilitates microbial communication, and retains nutrients. Consequently, novel strategies to counteract biofilms are under investigation. Fatty acids have emerged as promising prebiotic agents, defined as substances that stimulate the growth of beneficial bacteria. These compounds can disrupt biofilm structure and increase microbial susceptibility to treatment. Short- and medium-chain fatty acids demonstrate direct antimicrobial activity and can alter microbial community composition, thereby inhibiting biofilm formation in several pathogens, including oral species. For instance, omega-3 fatty acids effectively inhibit *Staphylococcus aureus* and *Pseudomonas aeruginosa* biofilms through membrane disruption and quorum sensing (QS) inhibition. Additionally, long-chain fatty acids, particularly omega-3 and omega-6 polyunsaturated fatty acids, exhibit anti-inflammatory and antibacterial properties. This review synthesises current evidence on fatty acids as prebiotics, emphasising their mechanisms of action and therapeutic potential against drug-resistant biofilm-associated infections. Given the increasing prevalence of antimicrobial resistance, unsaturated and essential fatty acids rep-resent promising candidates for innovative biofilm-control strategies.

## 1. Introduction

The resilience of biofilms against antimicrobial agents and their persistent adherence to both biological and non-biological surfaces present significant challenges in healthcare and food processing settings. Microorganisms embedded within biofilm matrices exhibit altered physiological states, reduced metabolic activity, and heightened stress responses, which together enhance resistance to conventional antibiotics and disinfectants. Consequently, biofilm-associated infections often become chronic, recurrent, and difficult to eradicate, thereby significantly contributing to the global burden of antimicrobial resistance (AMR) [1,2,3].

The reduced effectiveness of conventional antimicrobials has intensified interest in alternative or complementary strategies to prevent biofilm formation or disrupt established biofilms without fostering resistance. In this context, naturally occurring compounds with multifunctional biological activities have garnered significant attention [4]. Fatty acids are particularly promising due to their structural diversity, broad-spectrum antimicrobial properties, and capacity to interfere with key biofilm-related processes [5,6]. Prebiotic short-chain fatty acids function as potent quorum-sensing (QS) inhibitors, suppressing signals that regulate biofilm formation and maintenance. Medium-chain and long-chain fatty acids, primarily derived from dietary sources, have also demonstrated antimicrobial and antibiofilm activities, often attributed to their ability to disrupt microbial membranes and interfere with essential signalling pathways [5,6,7,8,9,10,11]. Comparative analyses of these fatty acids and established quorum inhibitors elucidate the relationship between chain length and antibiofilm efficacy, suggesting a unifying mechanism underlying their effectiveness.

Despite the growing body of research on fatty acids as antimicrobial and antibiofilm agents, the literature remains fragmented and conceptually ambiguous. Distinctions among chain-length-dependent mechanisms, prebiotic versus bioactive effects, and indirect versus direct antibiofilm actions are frequently inadequately addressed. Furthermore, translational challenges related to formulation, delivery, and in vivo efficacy remain underexplored.

This review offers a critical and integrative synthesis of the role of fatty acids in biofilm control, with particular emphasis on chain-length-dependent mechanisms, microbiota-related effects, and translational limitations. The objective is to clarify current knowledge gaps and propose future research directions for the strategic application of fatty acids as antibiofilm agents.

The narrative review was conducted through systematic searches of PubMed, Scopus, and Web of Science for peer-reviewed articles published up to 2025. Search terms included combinations of “fatty acids”, “short-chain fatty acids”, “medium-chain fatty acids”, “long-chain fatty acids”, “polyunsaturated fatty acids”, “prebiotic”, “biofilm”, “antibiofilm activity”, “quorum sensing”, and “microbiota”. Additional relevant studies were identified by manually screening the reference lists of key articles.

## 2. Fatty Acids as Prebiotics: Conceptual Boundaries and Critical Perspectives

The International Scientific Association for Probiotics and Prebiotics (ISAPP) has updated the consensus definition of a prebiotic to “a substrate that is selectively utilised by host microorganisms to confer a health benefit.” This revision broadens the concept to potentially include non-carbohydrate compounds [12,13]. The updated definition emphasises the functionality of selectively fermented ingredients that induce specific changes in gastrointestinal microbiota composition or activity, thereby conferring benefits to the host [14,15,16,17,18,19]. This expanded perspective allows for the inclusion of compounds with diverse chemical structures as prebiotics, provided they are selectively utilised by the microbiota and confer a health benefit [2,20]. A substance is classified as a prebiotic if it possesses a defined structure and composition, is selectively utilised by the host microbiota, results in measurable modulation of the microbiome, and demonstrates a health benefit. These criteria distinguish prebiotics from other compounds that may influence the microbiota but lack selectivity or specific health benefits. Furthermore, a prebiotic must be resistant to gastric acidity, mammalian digestive enzymes, and gastrointestinal absorption, ensuring it remains intact until reaching the colon for fermentation by the intestinal microflora. Selective fermentation alters the composition or activity of the intestinal microbiota, thereby conferring health benefits to the host [21]. These benefits include supporting probiotics, which are essential for establishing and maintaining a favourable environment for beneficial gut microbiota. Distinguishing the functional differences among short-, medium-, and long-chain fatty acids is essential for accurately delineating their prebiotic potential, as these molecules exert distinct effects on the gut microbiota and host physiology. Clarifying these distinctions prevents conceptual ambiguity and semantic inflation of the term “prebiotic.” A more rigorous and biologically consistent framework should classify SCFAs as microbiota-derived signalling metabolites, while MCFAs and LCFAs should be considered bioactive dietary lipids with secondary microbiota-modulating properties. Recognising these boundaries is critical for accurate interpretation of experimental data and for guiding future mechanistic and translational research in this field [22]. SCFAs, which contain fewer than six carbon atoms and are primary metabolites of microbial fermentation of indigestible dietary fibre, play a critical role in modulating both intestinal and systemic health. Acetate, propionate, and butyrate exert prebiotic effects by selectively stimulating the growth of beneficial bacteria and inhibiting pathogenic species, a process also mediated by the reduction in caecal pH resulting from their production [23]. These fatty acids are key regulators of the host immune system, influencing processes such as phagocytosis, chemokine production, and cell signalling pathways [24]. Notably, butyrate is essential for intestinal health and immune regulation, serving as both an energy source for colonocytes and a signalling molecule with anti-inflammatory properties [25,26]. In contrast, MCFAs and LCFAs, including PUFAs, which are primarily obtained from the diet, do not meet the strict ISAPP criteria for prebiotics. However, increasing evidence suggests that these fatty acids can indirectly modulate gut microbial ecosystems through mechanisms such as antimicrobial pressure, competitive exclusion, and host-mediated effects, thereby shifting microbial composition and metabolic activity. These effects represent ecological restructuring rather than selective microbial utilisation and therefore do not align with the classical definition of prebiotic activity. Dietary fatty acids play significant roles in energy metabolism and influence the microbiota and intestinal physiology by modulating intestinal permeability and exerting anti-inflammatory activity, rather than through direct fermentation [27]. Although MCFAs and LCFAs are not conventionally classified as prebiotics, they exert indirect effects on gut microbiota composition and activity. MCFAs can modulate intestinal permeability and inflammation, thereby altering the environment for resident microorganisms and exhibiting direct antimicrobial properties that influence gut microbial composition [28]. LCFAs primarily support nutrient absorption and host energy metabolism and may also indirectly affect the microbiota by altering substrate availability and intercellular signalling. While these effects do not directly stimulate beneficial microbial species, the promotion of beneficial gut bacteria and the inhibition of harmful species [29] foster a healthier gut environment and enhance the production of beneficial metabolites, such as SCFAs [30]. These metabolites are vital for maintaining colonic epithelial health, strengthening the gut barrier, modulating immune response, and regulating systemic metabolism [31]. By being selectively utilised by beneficial microorganisms, these compounds contribute to bacteriocin production and inhibit pathogenic bacteria, thereby supporting a healthier gut microbiome [32]. Certain PUFAs, such as eicosapentaenoic acid (EPA) and docosahexaenoic acid (DHA), exhibit anti-inflammatory and anti-diabetic properties, effects that are partly mediated by interactions with the gut microbiome [16,20,33]. These interactions demonstrate that fatty acids can function both as direct modulators of microbial communities and as indirect contributors to host systemic health through microbiome-mediated pathways [2]. Additionally, these fatty acids can alter the gut environment by modifying pH and oxygen levels, creating conditions less favourable for dysbiotic bacteria [34,35,36]. Notably, n-3 PUFAs restore eubiosis after dysbiosis and enhance SCFA production by acting as prebiotics for specific bacterial families, such as *Bacteroidetes* and *Lachnospiraceae* [23,37,38]. In individuals with inflammatory bowel disease (IBD), n-3 PUFA supplementation has been observed to restore a healthier microbiota composition, characterised by decreases in *Faecalibacterium* and increases in *Lachnospiraceae* and *Bacteroidetes* [23,38,39,40]. The effectiveness of omega-3 PUFAs appears to be dose-dependent, with studies indicating that higher doses (e.g., 60–90 mg in animal models) may be required to fully restore certain microbial taxa [41]. Human trials have employed various dosages, including 5 g/day of fish oil providing 1.9–2.2 g EPA and 1.1 g DHA [42], or DHA-enriched canola oil [37]. Although the sample sizes of studies demonstrating these direct changes in IBD patients vary, systematic reviews synthesising these findings suggest general trends [39]. However, some comprehensive meta-analyses of randomised controlled trials (RCTs), including studies with up to 69 RCTs, have reported little or no significant impact of omega-3 supplementation on IBD treatment or inflammatory status. This highlights potential study heterogeneity and underscores the need for further targeted research [43]. Table 1 summarises the definition of prebiotics and the mechanisms of action of fatty acids as prebiotics.

### 2.1. Short-Chain Fatty Acids (SCFAs) as Prebiotics

Alterations in microbial composition, especially the proliferation of beneficial taxa and modifications in their metabolic activities, illustrate the intricate relationship between dietary fatty acids and the gut microbiome in both health and disease.

Among lipids, n-3 PUFAs, particularly those derived from marine sources, significantly modulate the gut microbiota. They increase beneficial bacteria, such as *Lactobacillus*, *Lachnospiraceae*, *Bacteroidetes*, and *Roseburia*, while reducing *Faecalibacterium* and *Barnesiella*. These changes promote butyrate production and gut homeostasis and attenuate inflammatory responses [23,38,42,43,44,45]. The evidence indicates that n-3 PUFAs possess substantial prebiotic potential, particularly in enhancing beneficial microbial populations and maintaining a balanced gut environment [46]. In animal models, marine lipids and n-3 PUFAs also promote the growth of Bifidobacteria and commensal lactic acid bacteria, thereby supporting gastrointestinal stability even under high-fat dietary conditions [47]. Alterations in gut microbiota composition are linked to increased production of short-chain fatty acids, especially butyrate, and reduced levels of pro-inflammatory mediators, which contribute to systemic health benefits [47,48,49]. These findings underscore the integral role of the gut microbiota in mediating the systemic effects of n-3 PUFAs, particularly in metabolic regulation and inflammation [50]. For instance, omega-3 PUFA supplementation increases the abundance of *Bacteroides* and *Coprococcus* species while reducing *Collinsella* species, which are linked to fatty liver, thereby demonstrating a direct effect on gut microbial populations [51]. The method of n-3 PUFA administration can also significantly affect the microbiota, as certain functional beverages exert a greater influence on butyrate-producing bacterial genera [52]. Furthermore, conjugated linoleic acid (CLA) and monounsaturated fatty acids exhibit notable prebiotic potential by selectively promoting beneficial gut bacteria and inhibiting pathogenic strains [53]. Fatty acids also support the repair of compromised intestinal mucosa [54]. Recent in vitro models, such as the Mucosal SHIME (Simulator of the Human Intestinal Microbial Ecosystem), indicate that ω-3 PUFAs primarily modulate mucolytic species, such as *Akkermansia muciniphila*, increasing their abundance and enhancing their metabolic activities [55]. Collectively, these findings demonstrate the substantial impact of n-3 PUFAs on the gut microbiome, particularly their capacity to enhance beneficial microbial populations and metabolic outputs, which is critical for maintaining intestinal and systemic health [56,57,58].

### 2.2. Medium- and Long-Chain Fatty Acids with Prebiotic Potential

Medium-chain fatty acids, such as those found in coconut oil, inhibit harmful bacteria and promote beneficial species. Long-chain fatty acids also counteract pathogens, modulate immune responses, and influence the gut microbiome, thereby helping to prevent inflammation [23]. Their interactions with gut bacteria involve alterations in fat-derived signalling molecules, such as oxylipins, which can either induce or reduce inflammation [59]. Conjugated linoleic acids further exemplify this complexity: cis-9, trans-11 CLA reduces inflammation by modulating immune cells, whereas trans-10, cis-12 CLA may exacerbate inflammatory responses [60].

Oxylipins serve as key modulators of the gut microbiome. Omega-6-derived oxylipins, including those from linoleic, linolenic, arachidonic, 5-hydroxyeicosatetraenoic acid (5-HETE), and adrenic acids, as well as plasma omega-3-derived oxylipins, are negatively associated with *Sutterella.* Oxylipins derived from arachidonic acid also show a negative correlation with Proteobacteria. Both omega-3 and omega-6 oxylipins regulate intestinal alkaline phosphatase, an enzyme that degrades bacterial toxins [59]. The ratio of omega-6 to omega-3 oxylipins is inversely associated with *Clostridium* cluster IV and *Butyricimonas* [59]. These findings indicate that dietary fats indirectly support gut health and modulate inflammation through selective and complex interactions with gut microbes, suggesting that dietary oxylipins may facilitate targeted interventions. The distinct effects of omega-3- and omega-6-derived oxylipins further underscore their opposing roles in gut ecology and disease risk [59,61]. Diets high in omega-3 fatty acids promote the growth of beneficial bacteria, such as *Bacteroidetes* and *Firmicutes*, while reducing populations of pro-inflammatory species. Conversely, omega-6-derived oxylipins from linoleic and dihomo-γ-linolenic acids are negatively associated with *Acidaminococcus* and *Phascolarctobacterium*. Increasing omega-3 intake and reducing omega-6 consumption may beneficially modulate the gut microbiota, metabolism, and immune function [59]. Elucidating the effects of lipid mediators on bacterial growth and activity is essential for developing precision nutrition strategies to address dysbiosis and inflammation [62,63,64]. Table 2 provides a summary of the effects of fatty acids, particularly n-3 polyunsaturated fatty acids (PUFAs), on gut microbiota and metabolic outcomes.

## 3. Biofilm

A biofilm is a structured microbial community embedded within a self-produced matrix that adheres to biotic or abiotic surfaces [65]. This three-dimensional network, which may be homogeneous or heterogeneous [66], consists of extracellular polymeric substances, including exopolysaccharides, proteins, lipids, and nucleic acids, collectively referred to as the matrixome [67]. The matrix imparts mechanical stability, facilitates adhesion, and protects against host defences and antimicrobials [68,69], resulting in emergent properties such as enhanced surface attachment, structural heterogeneity, and antimicrobial tolerance [67,70]. The structural and biochemical characteristics of the matrixome confer additional emergent properties, including increased surface adhesion, spatial and chemical heterogeneity, and pronounced antimicrobial recalcitrance [67]. Consequently, biofilm-associated microbes are distinguished from their planktonic counterparts by their ability to acquire resources and persist in hostile environments [70,71].

**Figure 1:**
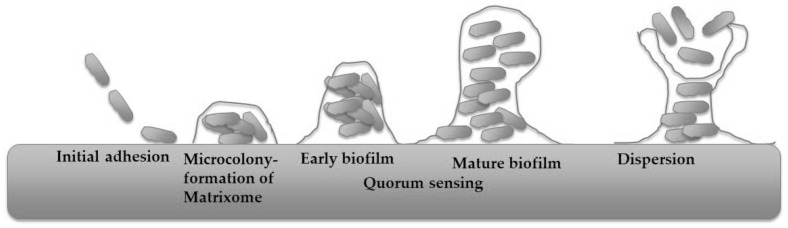
Typical process of bacterial biofilm development (modified from [71]).

The typical process of bacterial biofilm development is illustrated in Figure 1.

The process begins with the reversible attachment of planktonic cells, which is influenced by substrate characteristics such as roughness and hydrophobicity, as well as by hydrophobic, electrostatic, and Lifshitz–van der Waals forces. These factors complicate catheter sterilisation and present challenges for pipeline maintenance. Cellular appendages, including flagella, pili, fimbriae, and curli, also contribute to this initial attachment [72,73,74]. As cells synthesise extracellular polymeric substances (EPS), attachment transitions to an irreversible state, facilitated by adhesins such as collagen-binding proteins, lipopolysaccharides, and type IV pili [75,76]. Once stable attachment is established, cells proliferate, migrate across the surface, and form microcolonies, particularly under elevated cyclic di-GMP concentrations [77]. After microcolony formation, the maturation stage ensues. During this phase, microcolonies expand into macrocolonies, accompanied by increased production of EPS. This expansion produces a complex architecture with nutrient and waste channels that support metabolic activity [78,79]. Matrixome components, including polysaccharides, proteins, extracellular DNA, and lipids, stabilise the structure and enhance resistance to environmental stressors [80,81]. The viscoelastic properties of amyloids and cellulose contribute to both structural rigidity and adaptability [82]. Quorum-sensing mechanisms enable coordinated behaviour and phenotypic heterogeneity, allowing subpopulations to adapt to nutrient and oxygen gradients [83,84]. Ultimately, dispersal of cells from the mature biofilm enables recolonisation of new niches, thereby completing the biofilm life cycle [85,86].

### 3.1. Fatty Acids as Antibiofilm Agents

Fatty acids represent a promising strategy for antibiofilm therapy, as they can enhance the efficacy of existing antibiotic treatments and reduce bacterial resistance. These compounds function as multifunctional biofilm disruptors, effectively dismantling both bacterial and fungal biofilms. Unsaturated fatty acids, such as oleic acid and linoleic acid, demonstrate efficacy against diverse bacterial biofilms by disrupting initial cellular attachment or promoting biofilm dispersal [87,88]. Their broad-spectrum antibiofilm activity against various microbial pathogens supports their potential application in clinical infection management [6]. Certain fatty acids, including cis-2-decenoic acid (C2DA), serve as signalling molecules that induce biofilm dispersal and sensitise persister cells to antimicrobial agents, thereby expanding their functional scope beyond direct eradication [89]. This dispersal often occurs at sub-inhibitory concentrations, where fatty acids modulate cellular communication pathways or directly affect the biofilm matrix without inhibiting bacterial proliferation [89]. Omega-3 PUFAs have attracted considerable attention due to their diverse biological activities, particularly their potential as antibiofilm agents and disruptors of the extracellular matrixome [90,91]. Their amphipathic structure facilitates membrane disruption [92]. Although the development of conventional antibiotics initially overshadowed research into the antimicrobial properties of fatty acids, the rise in antibiotic resistance has renewed interest in unsaturated fatty acids as alternative or adjunct antimicrobial agents [93,94,95]. This renewed focus is supported by evidence that fatty acids can penetrate biofilm protective barriers, which often contribute to the reduced efficacy of standard antibiotics [22]. Monounsaturated and polyunsaturated fatty acids interact directly with bacterial membranes, compromising membrane integrity and inducing cell lysis, a process essential for accessing biofilm-embedded cells [95]. In addition to direct lysis, certain fatty acids act as diffusible signal factors in bacterial communication, promoting biofilm dispersal and inhibiting biofilm formation across diverse species [89]. These properties establish fatty acids as promising candidates for innovative antibiofilm therapies, particularly due to their ability to act synergistically with conventional antibiotics. This synergy arises from increased membrane permeability and alters efflux activity, which enhance intracellular antibiotic uptake. For example, myristoleic acid increases the antibiofilm efficacy of aminoglycoside antibiotics by promoting their penetration into the biofilm matrix, likely due to its surfactant-like properties [96]. Recent studies indicate that combining unsaturated fatty acids, such as palmitoleic acid and linoleic acid, with vancomycin leads to significant reductions in both methicillin-sensitive and methicillin-resistant *Staphylococcus aureus* (MRSA) populations, including antibiotic-tolerant cells [97]. Certain fatty acids can overcome multidrug resistance in pathogens such as *Pseudomonas aeruginosa* by targeting key virulence factors and biofilm-forming pathways, which is particularly valuable for addressing persistent infections [22]. Research also demonstrates that alpha-linolenic acid, a precursor to longer-chain omega-3 fatty acids, disrupts *P. aeruginosa* biofilm formation and reduces virulence factor production [22]. Synthetic unsaturated fatty acids have also shown selective antibacterial activity against MRSA [98]. The potential of fatty acids as antibiotic adjuvants extends beyond their direct antimicrobial effects, as they can enhance the efficacy of existing antibiotics and provide a strategic advantage in combating antimicrobial resistance [6]. Their biocompatibility is essential for clinical application, whether used as standalone agents or as enhancers of current antimicrobials, particularly given the significantly reduced susceptibility of biofilms to antibiotics compared to planktonic cells. Investigating fatty acids as antibiotic adjuvants is therefore a pivotal strategy for improving the effectiveness of conventional treatments against resistant pathogens and persistent biofilms. Recent research on omega-3 PUFAs, such as DHA, has confirmed their safety and efficacy as antibiofilm agents against pathogens, including *S. aureus* and MRSA, without inducing *ica*-ADBC-dependent biofilm formation or stress responses that could increase antibiotic resistance [91,99].

#### 3.1.1. Inhibition of Initial Adhesion and Early Biofilm Development

Initial adhesion represents a critical stage targeted by fatty acids during early biofilm formation. Fatty acids influence bacterial surface charge, hydrophobicity, flexibility, and adhesin expression, which reduces attachment to both biotic and abiotic surfaces [22]. The primary mechanism is the incorporation of fatty acids into the bacterial outer membrane, altering membrane permeability and fluidity and impeding stable surface colonisation. Essential and unsaturated fatty acids, including EPA, DHA, and gamma-linolenic acid (GLA), demonstrate strong inhibitory effects on adhesion and early biofilm development, particularly in vancomycin-resistant *Enterococcus faecium* [6]. Oleic, palmitic, and linoleic acids, which are prevalent in various plant oils, modulate pathogen–probiotic ratios and suppress early biofilm formation [88]. Omega-3 fatty acids, such as EPA and DHA, further reduce bacterial adhesion by modifying cell-surface properties, thereby making attachment and community formation less favourable. These effects are especially notable in *Streptococcus mutans*, where EPA and DHA significantly decrease biofilm thickness [95].

#### 3.1.2. Membrane Disruption and Detergent-like Activity

Many fatty acids, particularly unsaturated fatty acids, exert direct bactericidal and antibiofilm effects through detergent-like interactions with bacterial membranes. This mechanism is widely regarded as a primary target of fatty acid action [93,94]. The amphipathic nature of these molecules enables them to destabilise membrane lipids by inserting into the lipid bilayer [92], which increases membrane permeability and fluidity [100,101,102]. As a result, vital intracellular contents, such as ions, adenosine triphosphate (ATP), proteins, and nucleic acids, leak from the cell [93,94], ultimately leading to cell lysis [92,94]. Disruption of membrane integrity alters bacterial morphology and prevents cells from sustaining metabolic processes. The resulting loss of electron transport chain function and uncoupling of oxidative phosphorylation further impairs cellular energy production [93,94]. This process is essential for accessing and eliminating biofilm-embedded cells, which are typically protected by the EPS matrix. For example, the bactericidal effect of fatty acids derived from *Hermetia illucens* larvae fat against *Klebsiella pneumoniae* has been associated with increased bacterial outer membrane permeability [92]. Oleic, linoleic, and palmitoleic acids significantly inhibit biofilm growth in *S. epidermidis*, *S. aureus*, and *S. mutans* by compromising membrane integrity and disrupting cellular homeostasis [95]. Unsaturated fatty acids, including linoleic and arachidonic acids, generally demonstrate greater efficacy than their saturated counterparts by interfering with peptidoglycan and fatty acid synthesis pathways.

#### 3.1.3. Disruption of the EPS Matrix

Fatty acids compromise mature biofilms by directly targeting and disrupting the extracellular polymeric substance (EPS) matrix, also known as the matrixome. The EPS matrix is a complex mixture of polysaccharides, proteins, extracellular DNA (eDNA), and lipids that encases bacterial cells and confers structural stability, protection against environmental stressors, and resistance to antibiotics [103,104]. Fatty acids disrupt the integrity of this matrix through several mechanisms:

Modulation of EPS Biosynthesis: Ginkgolic acid may inhibit and disrupt biofilms by affecting the expression of genes involved in exopolysaccharide biosynthesis. Although the precise molecular mechanisms remain incompletely characterised, existing studies indicate that fatty acids can modulate gene expression [105].

Weakening of Matrix Structure: Oleic and linoleic acids reduce cell-surface hydrophobicity, thereby weakening nascent biofilms and decreasing bacterial attachment to host tissues and abiotic surfaces [6,96]. This change in surface properties impedes the formation and maintenance of the EPS network.

Direct Degradation of Matrix Components: Both omega-3 and omega-6 polyunsaturated fatty acids are believed to impair EPS integrity and promote matrix degradation [87]. Specifically, EPA and DHA degrade essential matrix components, such as polysaccharides, proteins, and extracellular DNA, in biofilms of *S. mutans*, *Porphyromonas gingivalis*, and *Fusobacterium nucleatum* [95]. This degradation reduces the biofilm’s biomass and the viability of embedded cells.

Disruption of Structural organisation: Gamma-linolenic acid downregulates key genes in mature vancomycin-resistant *E. faecium* biofilms, disrupting their structural organisation [6]. This results in a less cohesive and more vulnerable biofilm structure.

Consequently, omega-3 PUFAs demonstrate broad-spectrum antibiofilm activity in oral, staphylococcal, and mixed-species biofilms [90,99].

#### 3.1.4. Modulation of Quorum Sensing and Biofilm Dispersal

Several fatty acids disrupt quorum-sensing (QS) networks that control biofilm maturation and virulence. QS is a density-dependent mechanism in which bacteria communicate through small, diffusible signal molecules, known as autoinducers, to regulate gene expression for processes such as biofilm formation, virulence factor production, and stress tolerance [106,107,108,109]. Fatty acids function as quorum quenching agents [110], directly interfering with these signalling systems to inhibit biofilm establishment or facilitate its breakdown.

Suppression of QS-Regulated Genes: Linolenic acid suppresses QS-regulated biofilm formation in *P. aeruginosa* and reduces the expression of virulence genes [22].

Induction of Biofilm Dispersal: Cis-2-decenoic acid (C2DA), a diffusible signal factor produced by bacteria, induces biofilm dispersal at nanomolar concentrations without inhibiting bacterial growth. This process promotes the transition to planktonic cells and increases antibiotic susceptibility [89,93]. Such interference may involve inhibition of specific enzymes in the QS pathway or disruption of autoinducer production or detection, including N-acyl homoserine lactones (AHLs), which are prevalent in Proteobacteria [109,111].

Interference with Specific QS Systems: In *Acinetobacter baumannii*, unsaturated fatty acids may inhibit biofilm formation by blocking specific QS systems, thereby reducing virulence. Although the precise mechanisms of systems such as AbaIR are not fully described in the current literature, fatty acids are recognised to modulate virulence repression by inhibiting transcriptional activators [105].

Inhibition of QS by fatty acids disrupts the coordinated behaviours required for mature biofilm development, thereby increasing bacterial susceptibility to host defences and antimicrobial agents.

#### 3.1.5. Modulation of Gene Expression and Virulence Factors

**Figure 2:**
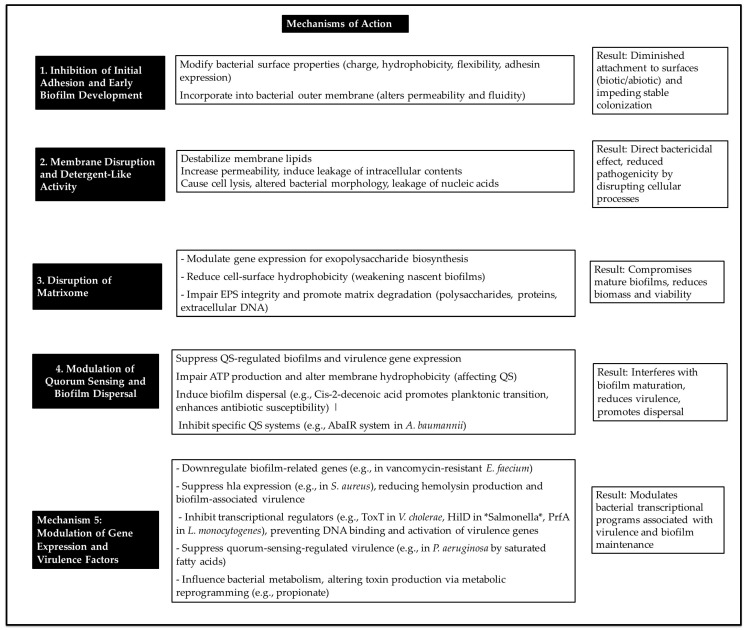
Main mechanisms of action of fatty acids against bacterial biofilms.

Beyond structural disruption, fatty acids modulate bacterial transcriptional programmes linked to virulence and biofilm maintenance. GLA significantly downregulates biofilm-associated genes in vancomycin-resistant *E. faecium* [6]. Fatty acids inhibit transcriptional regulators, thereby preventing DNA binding and the activation of virulence genes [105]. LCFAs act as allosteric inhibitors, reducing the DNA-binding affinity of transcriptional activators of virulence genes and modulating the activation of histidine kinase receptors. This modulation alters downstream intracellular signalling networks, resulting in the repression of virulence [105]. Fatty acids also influence bacterial metabolism; for example, propionate alters toxin production through metabolic reprogramming. Furthermore, alterations in fatty acid synthesis are associated with decreased expression of major virulence factors [112]. Figure 2 provides an overview of the mechanisms by which fatty acids function as antibiofilm agents.

#### 3.1.6. Spectrum of Activity and Therapeutic Relevance

Oils containing high levels of omega-3 fatty acids demonstrate significant efficacy against specific pathogens. For instance, herring oil and its omega fatty acids at 100 µg/mL reduce *S. aureus* MSSA 6538 biofilms by 75%, while 20 µg/mL achieves a 65% reduction in *S. aureus* MSSA33591 [113]. Saw palmetto oil exhibits activity against *S. aureus* biofilm at 20 mg/mL [96]. Multiple seed oils, particularly those rich in omega-3 fatty acids, inhibit biofilm formation by various pathogens. *Calophyllum* seed oil at 20 µL/mL inhibits biofilm formation by *A. baumannii* and *S. aureus* by 50.34% and 56.09%, respectively. Prickly pear seed oil inhibits *S. aureus* and *E. coli* biofilm formation by 63% and 80%, respectively [114]. Fratianni et al. reported that borage seed oil inhibited the metabolism of sessile cells, notably *L. monocytogenes* (54.4% inhibition) and *P. aeruginosa* (50.83%) [114].

Additional oils, such as coffee, pumpkin, and watermelon oils at 20 µL/mL, inhibit mature *S. aureus* biofilms by 59.22%, 57.39%, and 66.92%, respectively. At this concentration, broccoli seed oil and green coffee seed oil inhibit mature *L. monocytogenes* biofilms by 53.41% and 55.99%, respectively. Green coffee seed oil also inhibits mature *A. baumannii* biofilms by 53.72% [115].

Individual fatty acids exhibit potent antibiofilm activities at relatively low concentrations. For example, DHA at 0.612 and 1.25 mg/L modulates gene expression associated with biofilm production in *S. aureus* [91]. Both DHA and EPA completely inhibit biofilm formation and *P. gingivalis* growth at 12.5 μM and significantly reduce *F. nucleatum* biofilm formation at 100 μM [116]. At 100 μM, DHA and EPA also inhibit *S. mutans* biofilm growth [116]. Additionally, 125 µg/mL cis-2-decenoic acid (C2DA) disperses 40% of *P. aeruginosa* biofilm [89]. N-acylethanolamines, including oleoylethanolamide and anandamide at 64 mg/mL, inhibit *S. aureus* biofilms by reducing surface motility, aggregation, and the expression of biofilm-associated genes [116,117,118]. Certain fatty acids at 1.17 mg/mL can overcome multidrug resistance in pathogens such as *P. aeruginosa* by targeting key virulence factors and biofilm-forming pathways [119].

The low toxicity of certain fatty acids, such as cis-2-decenoic acid, at antibiofilm concentrations up to 620 nM, suggests a reduced risk of resistance development [120]. Synthetic unsaturated fatty acids with selective activity against MRSA further underscore the potential of this compound class as leads for next-generation antibiofilm and antibacterial agents [98]. The multimodal activities of fatty acids, including membrane disruption, matrix degradation, quorum-sensing inhibition, virulence repression, and synergy with existing antibiotics, support their promise as candidates for innovative antibiofilm therapies, particularly against multidrug-resistant infections [6,7,105,119,120,121,122]. Table 3 summarises mechanistic roles of fatty acids in biofilm control.

## 4. Specific Fatty Acids and Their Antibiofilm Effects

The structural diversity of fatty acids significantly influences their antimicrobial and antibiofilm activities. Key factors such as chain length, degree of saturation, and stereochemistry determine their capacity to disrupt bacterial membranes or interfere with quorum-sensing pathways [123]. Long-chain fatty acids (LCFAs) not only modulate bacterial functions but also serve as nutrient sources, underscoring their dual metabolic and therapeutic significance [105]. Several LCFAs inhibit *hil*A promoter activity by prevention of HilD binding to DNA, which reduces *Salmonella* virulence [105]. For instance, cis-2-hexadecenoic acid acts as a diffusible signal factor that binds to and inhibits HilD and modulates virulence gene expression in *S. enterica* [124]. Long-chain unsaturated fatty acids, such as cis-palmitoleate, also decrease HilD’s DNA-binding affinity and occupy a hydrophobic pocket in the N-terminal domain of ToxT, which alters its dimerization and DNA interactions [105,125]. Specific residues, including R267 and N44 in HilD, interact with fatty acid head groups and induce conformational changes that affect DNA binding [126]. The presence of a cis-2 unsaturation, characteristic of diffusible signal factors, is essential for regulatory activity and influences bacterial virulence, motility, and biofilm formation through quorum sensing [126]. Mono-PUFAs, such as palmitoleic and myristoleic acids, inhibit *tcp* gene expression in *Vibrio cholerae* by prevention of ToxT dimerization and promoter binding [109,127]. The targeted interactions demonstrate that LCFAs disrupt regulatory proteins to suppress virulence rather than bacterial growth, thereby limiting the development of resistance [124,126].

### 4.1. Butyrate and Its Role in Biofilm Inhibition

Butyrate (C4:0), a short-chain fatty acid, has garnered significant attention for its multifaceted roles in host physiology and potent antimicrobial and antibiofilm properties, primarily through modulation of the gut microbiota. Butyrate modulates host immunity and metabolism, directly influencing pathogen virulence and contributing to intestinal homeostasis [122]. Its antibiofilm activity arises from the regulation of bacterial gene expression and disruption of quorum-sensing pathways, which impedes biofilm formation and maturation. Additionally, butyrate acetylates lysine residues on transcriptional regulators, such as HilA in *Salmonella* SPI-1, leading to downregulation of virulence genes [128]. While this acetylation reduces virulence, it concurrently promotes biofilm formation in *S. enterica*, illustrating a complex interaction in which butyrate differentially modulates bacterial phenotypes based on gene targets and environmental context [128]. This paradox highlights an adaptive bacterial strategy within the host, in which diminished invasion may be compensated for by enhanced biofilm formation [122].

### 4.2. Propionate and Acetate in Biofilm Modulation

Beyond butyrate, the other two major SCFAs produced by the gut microbiota—propionate and acetate—also strongly influence bacterial biofilm formation and virulence. Propionate represses several *Salmonella* SPI-1 genes, including *hilA and hilD,* which reduces invasion by destabilisation of HilD and disruption of intracellular pH homeostasis [122]. Propionate and butyrate both inhibit *S. enterica* serovar *Typhimurium* biofilm formation in laboratory and food models, showing broad antibiofilm activity [129]. Acetate inhibits extracellular polysaccharide production and has anti-quorum-sensing effects in *E. coli,* reducing biofilm formation [122,130]. Although the molecular targets of acetate remain unclear, it is likely to interfere with autoinducer signalling [128]. Propionate also targets HilD directly, decreasing SPI-1 expression and limiting *S. enteritidis* invasion [131]. Notably, propionate and butyrate generally suppress *Salmonella virulence,* whereas acetate can enhance invasion gene expression in the distal small intestine at physiological concentrations [132].

### 4.3. Unsaturated Fatty Acids (UFAs) and Antibiofilm Activity

UFAs with defined chain lengths and structural features inhibit biofilm formation through disruption of signalling pathways, compromise of cell wall integrity, attenuation of efflux systems, and interference with quorum sensing. LCUFAs, such as oleic and linoleic acids, downregulate virulence genes including *hilA* and *hilD* in *S. Typhimurium*, which reduces bacterial invasion and dissemination [126,133,134,135]. Similar inhibitory effects are observed in other bacterial species. For instance, SCFAs produced by *Cutibacterium acnes*, such as propionic, isobutyric, and isovaleric acids, inhibit *S. epidermidis* biofilms through decreased exopolysaccharide production and increased antibiotic susceptibility via concentration-dependent mechanisms that modulate immune responses and reduce bacterial viability. Furthermore, short-chain fatty acid production by commensal *C. acnes* restricts the growth and biofilm formation of *S. aureus* [1,136]. SCFAs, including caproic and caprylic acids, also downregulate virulence genes such as *fim*A and *hil*A in *S. Typhimurium*, which diminishes its capacity to invade porcine intestinal epithelial cells [133].

### 4.4. Conjugated Linoleic Acid (CLA) and Biofilm Disruption

CLA and other SCFAs function as potent antibiofilm agents through disruption of bacterial membrane integrity and inhibition of EPS synthesis. Lipid-producing *L. casei* strains that enhance short-chain fatty acid (SCFA) output significantly modify the surface properties of *Salmonella* and *E. coli*, resulting in reduced hydrophobicity, decreased auto-aggregation, and diminished biofilm formation through interactions with the cytoplasmic membrane [137]. These changes lead to increased membrane permeability, leakage of intracellular contents, and impaired early adhesion, all of which are critical for biofilm development. SCFAs also modulate quorum-sensing–related gene expression, further inhibiting biofilm maturation [1]. At physiological concentrations, SCFAs suppress biofilm formation in several pathogens, including *S. epidermidis*, and frequently demonstrate greater efficacy than traditional antibiotics against established biofilms [1,137]. SCFA-overproducing *L. casei* strains markedly inhibit biofilm formation and reduce adhesion and invasion of *S. Typhimurium* and enterohaemorrhagic *E. coli* on INT-407 cells [137]. Genetically engineered *L. casei* strains with enhanced SCFA production confer even greater protection against enterohaemorrhagic *E. coli* (EHEC) growth and infection in both in vitro and in vivo models [138]. Table 4 summarises the effects of these fatty acids on specific bacterial biofilms.

## 5. Dosage and Toxicity Considerations

Fatty acids are generally regarded as safe dietary components; however, their antimicrobial and antibiofilm activities exhibit strong dose-dependency [23,153]. Concentrations required for effective biofilm inhibition frequently surpass physiological levels, especially for medium-chain and long-chain fatty acids (MCFAs and LCFAs) [38,44]. At higher doses, certain fatty acids may induce cytotoxicity, disrupt epithelial integrity, or trigger pro-inflammatory responses [45,46,47]. The efficacy and safety of fatty acids are influenced by microbial species, biofilm maturity, and environmental factors, emphasising the necessity of thorough evaluation of dosage, formulation, and delivery methods prior to translating in vitro results to clinical or industrial contexts [48,64].

### 5.1. Dose-Dependency of Antimicrobial and Antibiofilm Activity

Fatty acids such as stearic acid and oleic acid disrupt cell membranes in a concentration-dependent manner at levels above 1100 µM [155]. Saturated fatty acids, including myristic and palmitic acid, have been shown to reduce cellular growth rates in a dose-dependent fashion up to 250 µM and can induce apoptosis [156]. The minimum inhibitory concentration (MIC) is essential for determining the lowest effective concentration of MCFAs required to inhibit bacterial growth, demonstrating their dose-dependent antimicrobial activity against bacteria such as *E. coli*, *S. Typhimurium*, and *Campylobacter coli* [157]. Therefore, MIC determination is a prerequisite for subsequent assays, such as crystal violet and 3-(4,5-dimethylthiazol-2-yl)-2,5-diphenyltetrazolium bromide (MTT) or 2,3-bis-(2-methoxy-4-nitro-5-sulfophenyl)-2H-tetrazolium-5-carboxanilide (imethylthiazol-2-yl)-2,5-diphenyltetrazolium bromide (MTT) or 2,3-bis-(2-methoxy-4-nitro-5-sulfophenyl)-2H-tetrazolium-5-carboxanilide (XTT), to assess antibiofilm activity. Long-chain PUFAs also inhibit bacterial growth in a dose-dependent manner, with MICs against pathogens including *C. acnes* and *S. aureus* ranging from 32 to 1024 mg/L [93].

### 5.2. Concentrations Exceeding Physiological Levels and Cytotoxicity

At higher concentrations, certain fatty acids exhibit lipotoxic effects. For instance, palmitic acid at micromolar concentrations induces apoptosis in pancreatic cells, while some monounsaturated fatty acids reduce membrane fluidity and contribute to lipotoxicity [156,158]. Excessive dietary intake of lipids, including oleic and palmitic acids, can negatively impact the structure and mechanotransduction of intestinal cells in vitro. Bergen et al. reported that exposing HCT116 cells to oleic and palmitic acid at concentrations above 25 µM significantly decreased cell membrane fluidity, with marked actin cytoskeleton rearrangement observed at 25 mM and 100 mM, respectively [159]. These results suggest that concentrations above physiological levels may compromise epithelial integrity.

### 5.3. Dependence on Microbial Species, Biofilm Maturity, and Environmental Conditions

The antimicrobial efficacy of fatty acids varies according to bacterial species, as shown in MIC studies with multiple pathogens [115,157]. Environmental factors, especially pH, significantly affect the antimicrobial and antibiofilm activities of short-chain carboxylic acids. For example, acidic conditions at pH 4.5 enhance efficacy against *S. enterica* [160].

### 5.4. Importance of Evaluating Dosage, Formulation, and Delivery Strategies

Developing innovative drug-delivery strategies is crucial for improvement of biofilm penetration, promotion of dispersal, and achievement of synergistic bactericidal effects of antibiofilm agents, including fatty acids, in clinical applications [161]. The therapeutic potential of specific fatty acids, such as gamma-linolenic acid, for treating biofilm-associated infections highlights the necessity for comprehensive evaluation prior to clinical use [16]. Furthermore, thorough safety assessments are essential when considering fatty acids for therapeutic purposes.

## 6. Clinical and Therapeutic Implications

The antibiofilm and immunomodulatory properties of fatty acids offer promising avenues for the development of novel therapeutics that target persistent bacterial infections. Linoleic acid-overproducing *L. casei* alters key physicochemical properties, such as hydrophobicity and auto-aggregation, in pathogens including *Salmonella* and *E. coli*, which highlights its potential for probiotic use. These engineered strains significantly reduce bacterial surface hydrophobicity and auto-aggregation, thereby interfering with adhesion mechanisms critical for biofilm formation [137]. Increased linoleic acid synthesis by *L. casei* induces both structural and functional damage to bacterial cell membranes, which leads to inhibited growth and reduced virulence of enteric pathogens. Furthermore, linoleic acid-overproducing *L. casei* decreases the attachment of *Salmonella* and enterohaemorrhagic *E. coli* to host cells, which demonstrates a direct impact on pathogen-host interactions [137].

Although CLA is often associated with beneficial effects on metabolism and inflammation, and its interactions with the gut microbiome are under investigation, direct evidence from human clinical trials that supports CLA as a prebiotic remains limited. Some studies suggest an indirect association; for example, a prebiotic dietary fibre intervention in obese patients increased faecal rumenic acid (a form of CLA), which correlated with the presence of *Bifidobacterium* [162]. This finding indicates that other prebiotics can influence CLA levels, which, in turn, are associated with beneficial bacteria. Another study assessed the impact of consumption of 1 L/day of cows’ milk containing 5 mg/g fat cis-9, trans-11 CLA, as well as higher concentrations of cis-9, trans-11 CLA and trans-10, cis-12 CLA for 8 weeks, on the human faecal microbiological profile. The results showed that CLA intake altered faecal microbiota composition [163]. However, these findings do not conclusively establish CLA as a direct prebiotic substrate for specific beneficial bacteria. The ability of the human gut microbiota to synthesise CLA from linoleic acid is still under investigation, with current evidence suggesting that this process is less well-characterised in humans than in vitro or animal models [164]. Several human trials involving CLA focus on its anti-obesity, metabolic, and anti-inflammatory properties, with changes in gut microbiota considered secondary outcomes rather than evidence of CLA functioning as a prebiotic [165,166]. For instance, Smedman and Vessby studied 53 healthy adults whose diets were randomly supplemented with CLA (4.2 g/day) or an equivalent amount of olive oil for 12 weeks in a double-blind design. The CLA-treated group experienced a 3.8% reduction in body fat that represented a significant difference compared to the control group [165]. In contrast, other fatty acids, particularly omega-3 PUFAs such as DHA and EPA, have shown promising in vitro antimicrobial and antibiofilm activities against various pathogens [6,91]. However, these findings are primarily based on laboratory or animal studies. To date, no human clinical trials have evaluated CLA as an antibiofilm agent for the treatment or prevention of biofilm-associated infections.

### 6.1. Fatty Acids in Gut Health Management

Targeted modulation of the gut microbiota using specific fatty acids constitutes an effective approach to enhance host defences and reduce gastrointestinal pathogen colonisation. This strategy is exemplified by probiotics such as *L. casei,* which can be engineered to overproduce beneficial fatty acids, including conjugated linoleic acid, thereby increasing their protective efficacy against enteric pathogens [137]. The cell-free supernatant from linoleic acid-overproducing *L. casei* significantly inhibits biofilm formation by enterohaemorrhagic *E. coli* and downregulates virulence genes, particularly those associated with type III secretion systems in *S. Typhimurium* and enterohaemorrhagic *E. coli* [137,138]. Suppression of these virulence factors restricts pathogen adhesion and invasion, which highlights the therapeutic potential of fatty acid-enriched probiotics [137].

### 6.2. Potential for Treating Biofilm-Related Infections

Fatty acids with demonstrated antibiofilm properties, such as CLA, offer promising alternatives or adjuncts to antibiotics in the context of rising multidrug resistance. Their capacity to disrupt established biofilms and inhibit initial microbial adhesion makes them strong candidates for antimicrobial strategies that bypass conventional resistance mechanisms. Probiotic strains, such as *L. casei,* can endogenously synthesise these bioactive fatty acids, reducing dependence on external antibiotics and lowering selective pressures that contribute to resistance. This endogenous production limits pathogenic biofilm formation, reinforcement of gut barrier integrity, and enhancement of host resilience [137]. Fatty acid metabolites further modulate inflammation by downregulating pro-inflammatory pathways and promoting anti-inflammatory mediators, thereby supporting gut and immune homeostasis. The combined immunomodulatory, antimicrobial, and antibiofilm effects of these metabolites position them as key regulators of host–pathogen interactions [137,152]. SCFAs also influence microbial growth, motility, biofilm development, and QS [153], thereby shaping microbial community composition and pathogenicity [154]. Additionally, CLA isomers can modulate immune responses and exhibit either pro-inflammatory or anti-inflammatory effects depending on their specific structure and biological context [150]. Clinical and therapeutic implications of fatty acids are summarised in Table 5.

## 7. Challenges and Opportunities in Therapeutic Applications

Despite their promising therapeutic potential, the clinical use of fatty acids as antibiofilm agents is limited by challenges in delivery, stability, and dose optimisation in complex physiological environments. Further research is required to clarify their mechanisms of action, including interactions with bacterial membranes and signalling pathways [167], thereby supporting the development of targeted strategies to disrupt or prevent biofilms. Exploring synergistic combinations of fatty acids with conventional antimicrobials may further enhance antibiofilm efficacy. Genetically engineering probiotic strains to boost fatty acid production and deliver them to specific host sites represents another promising avenue for personalised antibiofilm therapies. This aligns with the broader concept that free fatty acids can inhibit pathogens through multiple pathways without harming the gut microbiota [54]. Some fatty acids also help maintain skin microbial balance through their antimicrobial activity. For example, cis-2-decenoic acid induces biofilm dispersal at nanomolar levels and inhibits growth at higher concentrations [7]. When combined with conventional antibiotics, it shows enhanced antibiofilm effects [120], illustrating how coupling dispersal with growth inhibition offers a powerful strategy against persistent, biofilm-associated infections [7,120].

## 8. Future Directions and Research Gaps

Although numerous SCFAs demonstrate potent antibiofilm effects in vitro and in preclinical models, substantial gaps remain before translation of these findings into clinical practice. Comprehensive knowledge of their pharmacokinetics, bioavailability, and potential adverse effects in humans is required. Further research is also necessary to determine optimal dosing, delivery methods, and interactions with the host microbiome. Advanced encapsulation or targeted delivery approaches may enhance SCFA stability and bioavailability at infection sites. Additionally, the establishment of standardised protocols to evaluate antibiofilm activity across various bacterial species and biofilm architectures is critical [77]. Among SCFAs, C2DA is particularly promising and exhibits broad-spectrum activity against microorganisms such as *P. aeruginosa* and methicillin-resistant *S. aureus* [77,80]. This unsaturated fatty acid, produced by multiple bacterial species, primarily functions as a potent biofilm-dispersal signal and induces a transition from sessile to planktonic states without bactericidal effects at low concentrations [116,140]. At elevated concentrations, C2DA inhibits bacterial growth and prevents biofilm formation and demonstrates efficacy against *S. aureus* at 500 µg/mL and 125 µg/mL, respectively [77,80]. By dispersion of established biofilms, C2DA increases bacterial susceptibility to antimicrobials and facilitates synergistic effects with antibiotics or disinfectants that markedly reduce biofilm biomass [7,141]. Its capacity to function at nanomolar and environmentally safe concentrations, coupled with a low propensity for resistance development, underscores its potential for biofilm control in both industrial and clinical contexts [7]. This diminished risk of resistance further augments the effectiveness of prophylactic antibiotic regimens [77,140].

## 9. Comparison of Antibiofilm Strategies: Fatty Acids, Nanoparticles, Polyphenols, and Peptides

Several classes of molecules offer promising strategies for combating bacterial biofilms, each with distinct mechanisms and specific advantages (Table 5). Naturally occurring fatty acids disrupt bacterial membranes [92,93,94,95,102,155,168], inhibit bacterial adhesion, and modulate QS [89]. These compounds often act at subinhibitory concentrations and can enhance antibiotic efficacy [87,89,169].

Nanoparticles, engineered and biofabricated using micro- and nanotechnologies, are also used to combat biofilms. They can encapsulate and deliver antimicrobial agents [169,170,171,172], including natural compounds like carvacrol [170] or even fatty acids themselves (e.g., fatty acid-capped silver nanoparticles) [171], and provide an engineered approach for targeted and controlled release of antibiofilm agents, which improves biofilm penetration and reduces resistance development [172,173,174,175].

Polyphenols, natural plant-derived compounds, exert antibiofilm effects through multitarget mechanisms, including interference with QS and modulation of virulence factors, and show low potential to induce resistance [176,177,178,179,180].

Peptides, including antimicrobial and host defence peptides, permeabilise bacterial membranes, inhibit adhesion, and disaggregate the biofilm matrix. These molecules display broad-spectrum activity and a low propensity to induce resistance [181,182,183,184,185]. Together, these molecular classes offer complementary and potentially synergistic strategies to address the challenges posed by biofilm-associated infections.

Table 6 provides a comparative overview of these antibiofilm agents.

## 10. Limitations

Despite the considerable promise of fatty acids as antibiofilm agents, several limitations must be recognised when interpretation of the current evidence and evaluation of their potential for clinical application are considered.

### 10.1. Methodological Heterogeneity

Substantial methodological heterogeneity characterises the literature on fatty acid antibiofilm activity, which complicates direct comparisons across studies. Experimental designs vary across bacterial strains, biofilm cultivation methods (static versus flow systems), incubation durations, growth media composition, and biofilm maturity at the time of treatment. Additionally, diverse assessment methodologies—including crystal violet staining, metabolic assays (MTT, XTT), viable cell counts, and microscopic techniques—measure different aspects of biofilm integrity. This variability hinders the establishment of standardised efficacy thresholds and complicates meta-analytical synthesis. Adoption of standardised protocols, such as those recommended by international microbiology consortia, would facilitate more robust cross-study comparisons in future research.

### 10.2. In Vitro vs. In Vivo Discrepancies

Most of the evidence for fatty acid antibiofilm activity originates from in vitro studies conducted under controlled laboratory conditions. Whilst these models provide valuable mechanistic insights, they do not replicate the complexity of in vivo environments, where host immune responses, tissue architecture, blood flow, protein binding, and microbial community interactions significantly influence biofilm dynamics and antimicrobial efficacy. The limited in vivo studies, primarily in animal models, have produced variable results, making direct extrapolation to human clinical scenarios uncertain. Furthermore, the bioavailability, pharmacokinetics, and tissue distribution of fatty acids in vivo differ markedly from those in vitro, potentially impacting their antibiofilm efficacy at infection sites. The absence of clinical trials evaluating fatty acids as antibiofilm agents in humans represents a critical gap in translational research.

### 10.3. Dose Comparability and Concentration Issues

A major limitation involves comparability of effective concentrations reported across studies and their relevance to physiologically or clinically achievable levels. Many in vitro studies demonstrate antibiofilm activity at fatty acid concentrations exceeding those naturally present in biological fluids or attainable through dietary supplementation or pharmacological administration without adverse effects. For example, certain fatty acids exhibit potent antibiofilm effects at millimolar concentrations in vitro. However, achievement and maintenance of such levels systemically or at infection sites in vivo may be impractical or cytotoxic, as discussed in Section 5. Additionally, dose–response relationships are inconsistently reported, and minimum effective concentrations vary widely by bacterial species, biofilm age, and environmental conditions. This variability complicates the development of therapeutic dosing regimens and highlights the need for comprehensive pharmacokinetic and pharmacodynamic studies in relevant clinical settings.

### 10.4. Translational Constraints

Additional constraints hinder the immediate clinical translation of fatty acids as antibiofilm therapies. The stability and targeted delivery of fatty acids to infection sites are challenging due to their susceptibility to oxidation, enzymatic degradation, and rapid metabolism. Advanced formulation strategies, such as encapsulation, nanoparticle delivery systems, and prodrug approaches, are required but remain largely unexplored for antibiofilm applications. The potential for off-target effects, including changes in host lipid metabolism, modulation of inflammatory pathways, and impacts on commensal microbiota, necessitates thorough evaluation in long-term safety studies. Although fatty acids show synergistic effects with conventional antibiotics in vitro, optimal combinations, dosing regimens, and the risk of antagonistic interactions have not been systematically studied in clinical contexts. Furthermore, regulatory pathways for the approval of fatty acids as therapeutic agents, whether as standalone antimicrobials, adjuvants, or medical food ingredients, remain undefined and may differ across jurisdictions.

In summary, although current evidence supports the antibiofilm potential of fatty acids, substantial methodological, translational, and clinical gaps must be addressed before these compounds can be reliably incorporated into antimicrobial stewardship strategies. Future research should prioritise standardised methodologies, rigorous in vivo validation, pharmacokinetic optimisation, and well-designed clinical trials to facilitate the transition from promising preclinical findings to effective clinical applications.

## 11. Conclusions

Fatty acids represent a structurally diverse class of bioactive molecules with multifaceted roles extending from microbiota modulation to biofilm disruption. Their biological effects are highly dependent on chain length, degree of saturation, and concentration, resulting in distinct prebiotic-like, antimicrobial, and antibiofilm activities. While short-chain fatty acids primarily act as key mediators of host–microbiota interactions and demonstrate potent effects on quorum sensing and virulence gene expression, medium- and long-chain fatty acids exhibit more direct antibiofilm properties through membrane disruption, extracellular polymeric substance degradation, and interference with bacterial signalling pathways. The therapeutic potential of fatty acids is further enhanced by their synergistic interactions with conventional antibiotics, offering promising strategies to combat multidrug-resistant pathogens and persistent biofilm-associated infections. Their multimodal mechanisms of action—including modulation of bacterial adhesion, disruption of mature biofilms, and suppression of virulence factors—position them as valuable alternatives or adjuncts to traditional antimicrobials. The capacity of engineered probiotic strains to overproduce specific fatty acids adds another dimension to their clinical applicability, particularly in the management of gut health. However, as discussed in the preceding limitations section, significant knowledge gaps and translational challenges must be addressed before fatty acids can be reliably integrated into clinical practice. Methodological heterogeneity across studies, discrepancies between in vitro efficacy and in vivo performance, issues with dose comparability, and concerns about bioavailability and stability represent critical barriers to clinical translation. Future research should prioritise the development of standardised experimental protocols to enable robust cross-study comparisons and meta-analyses. Well-designed clinical trials are essential to establish optimal dosing regimens, evaluation of safety profiles, assessment of long-term effects, and validation of the efficacy of fatty acids in human biofilm-associated infections. Additionally, advancing delivery technologies—such as encapsulation systems, nanoparticle formulations, and targeted release mechanisms—will be crucial to enhance biofilm penetration and maintain therapeutic concentrations at infection sites while minimising systemic toxicity. Exploring synergistic combinations with other antibiofilm agents, including conventional antibiotics, antimicrobial peptides, and phytochemicals, may further amplify therapeutic efficacy and reduce the risk of resistance development. Elucidation of the precise molecular mechanisms underlying fatty acid antibiofilm activity, particularly their interactions with bacterial signalling networks and host immune responses, will enable rational design of next generation antibiofilm therapies. Ultimately, the successful translation of fatty acids from promising preclinical candidates to effective clinical interventions will require multidisciplinary collaboration among microbiologists, pharmacologists, clinicians, and pharmaceutical scientists. With continued rigorous investigation addressing the identified limitations, fatty acids hold substantial promise as complementary strategies against biofilm-associated infections in both clinical and food-related environments, contributing to the global effort to combat antimicrobial resistance.

## Figures and Tables

**Table 1:** Definition of prebiotics and mechanisms of action of fatty acids as prebiotics.

**Definition Expansion:**	Any substrate that is selectively utilised by host microorganisms to confer a health benefit is defined as a prebiotic, which moves beyond a carbohydrate-focused perspective [13].
	The ISAPP (International Scientific Association for Probiotics and Prebiotics) acknowledges non-carbohydrate molecules, such as fatty acids, for their capacity to modulate gut microorganisms and provide health benefits [16,17,18]. However, rigorous scientific validation is necessary to confirm non-omega-3 fatty acids as prebiotics [12,13,22].
	Certain fatty acids, including eicosapentaenoic acid (EPA) and docosahexaenoic acid (DHA), exhibit anti-inflammatory and anti-diabetic properties, partly mediated by interactions with the gut microbiome [22].
**Mechanisms of Action:**	Fatty acids function both as direct modulators of microbial communities and as indirect contributors to host systemic health via microbiome-mediated pathways [12,13].
	Fatty acids can alter the gut environment by alteration of pH and oxygen levels, which creates conditions less favourable for dysbiotic bacteria [38,39].
	Certain PUFAs found in fish oils promote beneficial gut bacteria whilst inhibiting harmful species [34,35,42].
	Selective modulation fosters a healthier gut environment and enhances the production of beneficial metabolites, such as SCFAs [42].
	Fermentation of fatty acids by gut microbiota produces SCFAs, which are vital for maintenance of colonic epithelial health and regulation of systemic metabolism [26].
	SCFAs contribute to bacteriocin production and inhibit pathogenic bacteria, thereby supporting a healthier gut microbiome [26].
**Specific Microbiota Effects:**	N-3 PUFAs restore eubiosis after dysbiosis and enhance SCFA production by action as prebiotics for specific bacterial families, such as Bacteroidetes and Lachnospiraceae [23,37,38,40].
	Fermentation of fatty acids by gut microbiota produces SCFAs, which are vital for maintenance of colonic epithelial health and regulation of systemic metabolism [26].
	SCFAs contribute to bacteriocin production and inhibit pathogenic bacteria, thereby supporting a healthier gut microbiome [26].

**Table 2:** Effects of fatty acids on gut microbiota and metabolic outcomes.

Fatty Acid Type/Source	Microbiota Modulation	Metabolic Effects	Physiological Outcomes
Dietary lipids (SFA, MUFA, PUFA)	General shifts in microbial composition; metabolic pathway modulation	Altered SCFA and bile acid production	Impact on gut and systemic health [58,63].
n-3 PUFAs (EPA, DHA; marine sources)	↑ *Lachnospiraceae, Bacteroidetes, Roseburia*; ↓ *Faecalibacterium*	↑ SCFAs, especially butyrate	Improved gut homeostasis; prebiotic-like effects [23,37,38,44,45,47,49].
n-3 PUFAs (animal models)	↑ *Bifidobacteria*; ↑ lactic acid bacteria under high-fat diets	↑ SCFAs	Prevention of GI dysregulation [47]
n-3 PUFAs (human trials)	↓ *Firmicutes/Bacteroidetes*; ↓ *Coprococcus, Faecalibacterium*; *↑ Bifidobacterium*, *Lachnospira, Lactobacillus*	↑ Butyrate; ↓ pro-inflammatory mediators	Metabolic and immunological benefits [47,48,62,63,64]
n-3 PUFA–microbiota metabolic interplay	Influence on microbial lipid biosynthesis	↑ SCFAs	Metabolic regulation and inflammation control [49,50]
Species-specific effects	↑ *Bacteroides, Coprococcus;↓ Collinsella*	–	Microbiota-mediated benefits [51]
Delivery form (functional beverages)	↑ Butyrate-producing genera	–	Matrix-dependent enhancement [52]
MUFA and CLA	Promotion of beneficial taxa; inhibition of pathogens	Improved barrier function	Anti-inflammatory potential [47,53]
Fish oil n-3 PUFAs	↑ *Barnesiella*, *Lactobacillus*, *Porphyromonadaceae*, *Bacteroidia*	–	Improved barrier integrity; ↓ inflammation [23]
FAT-1 mice	↑ *Clostridium* cluster IV (butyrate-producing)	↑ Butyrate	Anti-inflammatory intestinal environment [50]
M-SHIME in vitro model	↑ *A. muciniphila*	↑ Mucin-related metabolism	Enhanced mucosal health [54,55]
Omega-3-derived oxylipins	↑ *Clostridium* cluster IV	↑ β-oxidation of oxylipins	Reduced inflammation; improved barrier function [1,50,59,60]

**Table 3:** Mechanistic Roles of Fatty Acids in Biofilm Control.

Action	Fatty Acids	Mechanism	Targets
Initial adhesion inhibition	Oleic, linoleic, palmitic; EPA/DHA	Alter surface charge, hydrophobicity; reduce adhesion	*S. aureus, S. mutans, P. aeruginosa* [6,119]
Matrix disruption	EPA, DHA, oleic, palmitoleic	Membrane disruption; EPS degradation	*P. gingivalis, F. nucleatum* [8,76]
QS interference	ALA, EPA, DHA; palmitoleic	Inhibit AHL/QS regulators	*A. baumannii, P. aeruginosa* [119]
Virulence attenuation	LCFAs, MUFAs, SCFAs	Inhibit HilD, ToxT, PrfA	*Salmonella* sp., *Vibrio* sp. [105,122]
Biofilm dispersal	Cis-2-decenoic acid	DSF-mediated dispersal	*P. aeruginosa, K. pneumoniae* [7]

**Table 4:** Specific Fatty Acids and Their Antibiofilm/Antivirulence Effects.

Fatty Acid/Molecule	Category	Main Mechanism	Specific Antibiofilm/Antivirulence Effects
Cis-2-decenoic acid (C2DA)	Unsaturated FA, DSF-type signalling molecule	Induces biofilm dispersal; activates EPS-degrading enzymes; sensitises persisters to antibiotics	Disperses established biofilms and inhibits formation in *P. aeruginosa*, *E. coli*, *K. pneumoniae*, *S. aureus*, *B. subtilis*; enhances aminoglycoside activity [4,7,12,139,140]
Oleic acid	MUFA (long-chain)	Membrane insertion; alteration of cell-surface hydrophobicity; gene regulation (e.g., *hla*)	Inhibits *S. aureus* biofilm formation and haemolytic activity; decreases adhesion and virulence [6,9,141]
Linoleic acid	PUFA (ω-6)	Membrane and matrix disruption; QS interference; synergy with antibiotics	Inhibits early biofilm development; acts as adjuvant enhancing vancomycin killing; reduces virulence and adhesion in enteric pathogens [6,10,119,137,142]
α-Linolenic acid (ALA)	PUFA (ω-3)	QS disruption; interference with virulence factor production; modulation of fatty acid synthesis	Inhibits *P. aeruginosa* biofilm formation and virulence factor production; acts as potential immunomodulatory and antibiotic adjuvant [1,119,141]
Eicosapentaenoic acid (EPA)	Long-chain ω-3 PUFA	Membrane disruption; matrix degradation; QS modulation	Disrupts outer layers and matrix in *S. mutans* biofilms; reduces biomass and viability of *P. gingivalis* and *F. nucleatum*; antibiofilm activity against staphylococci and oral pathogens [8,76,91,119,142]
Docosahexaenoic acid (DHA)	Long-chain ω-3 PUFA	Membrane and matrix disruption; QS-related gene downregulation	Strong antibiofilm activity against *S. aureus*, MRSA and oral pathogens; reduces biofilm thickness and virulence without inducing icaADBC-dependent biofilms [76,91,142,143]
Palmitoleic acid	MUFA (C16:1)	QS inhibition (AbaIR system); membrane perturbation; synergy with antibiotics	Enhances vancomycin-mediated killing of *S. aureus* (incl. MRSA); reduces *A. baumannii* biofilms by downregulating *abaR* and AHL levels [10,11,91,144,145,146]
Myristoleic acid	MUFA (C14:1)	Membrane permeabilization; QS interference; antibiotic adjuvant	At 10 μg/mL, combined with tobramycin, reduces *S. aureus* biofilm survival by >4–5 log compared with tobramycin alone [9,10,12]
Ginkgolic acids	Alkylphenolic fatty acid-like compounds	Inhibition of EPS-related gene expression; membrane/matrix perturbation	Markedly inhibit and disrupt biofilms of *S. mutans* and *E. coli* O157:H7; affect exopolysaccharide production [16,92,93,94,95,100,101,147,148,149]
Butyrate (C4:0)	SCFA	Modulation of host and bacterial gene expression; lysine acylation (e.g., HilA); QS and SPI-1 regulation	Reduces *Salmonella* virulence via SPI-1 repression; paradoxically can promote biofilm formation in *S. enterica* while decreasing invasion [122,128,129]
Propionate	SCFA	destabilisation of HilD; intracellular pH modulation; SPI-1 downregulation	Inhibits *Salmonella* invasion and biofilm formation in vitro and in food models; represses SPI-1 genes *hilA/hilD* [122,129,131]
Acetate	SCFA	Inhibition of EPS production; anti-QS activity; possible interference with autoinducer signalling	Reduces biofilm formation in *E. coli* by decreasing extracellular polysaccharide production and QS; may enhance invasion gene expression in specific gut niches
Caproic and caprylic acids	Medium-chain fatty acids	Downregulation of virulence genes (e.g., *fimA*, *hilA*) and invasion factors	Reduce *S. Typhimurium* ability to invade porcine intestinal epithelial cells; decrease virulence [128,130,132,133]
Conjugated linoleic acid (CLA)	Conjugated PUFA (SCFA-like behaviour in context)	Membrane permeabilization; inhibition of EPS; modulation of QS-related genes	Strong antibiofilm activity; *L. casei* strains overproducing CLA reduce hydrophobicity, auto-aggregation and biofilm formation of *Salmonella* and EHEC; inhibit adhesion and invasion [137,138,150]
SCFAs from *Cutibacterium acnes*	Mix of propionic, isobutyric, isovaleric acids (SCFAs)	Reduction in EPS production and virulence; enhanced antibiotic susceptibility	Inhibit *S. epidermidis* and *S. aureus* biofilms; decrease exopolysaccharide production and increase susceptibility to antibiotics [1,136]
Long-chain UFA	LCFAs, many with cis-2 unsaturation	Direct interaction with regulators HilD, ToxT; occupation of hydrophobic pockets; conformational change and loss of DNA binding	Inhibit hilA promoter activity and attenuate *Salmonella* virulence; block ToxT in *V. cholerae*; affect motility and biofilm formation via QS [105,124,125,126]
Tetradecanoic acids and related LCFAs	LCFAs	QS interference and modulation of transcriptional regulators	Reduce pathogenic traits in *Proteus mirabilis*, *Chromobacterium violaceum*, *Vibrio* spp. and *P. aeruginosa* via QS and regulator inhibition [105,151]
Linoleic-acid-overproducing *L. casei* (cell-free supernatant)	Probiotic-derived fatty acid mixture (rich in CLA/linoleic derivatives)	Alteration of pathogen cell-surface properties; membrane damage; T3SS and virulence gene downregulation	Strongly inhibits biofilms and adhesion/invasion of *S. Typhimurium* and enterohemorrhagic *E. coli* on intestinal cells; protects in vitro and in vivo [137,138,152,153,154]

**Table 5:** Clinical and Therapeutic Implications of Fatty Acids.

Application	Fatty Acids	Effect	Targets
Antibiotic synergy	Myristoleic + tobramycin; palmitoleic + vancomycin	↑ permeability; persister killing	*S. aureus* (incl. MRSA) [9,10]
Probiotic enhancement	CLA, SCFAs (*L. casei*)	↓ adhesion/invasion; membrane damage	*Salmonella*, EHEC [137,138]
Gut microbiota modulation	Butyrate, propionate, acetate	↓ SPI-1 genes; ↓ virulence	*Salmonella*, *E. coli* [122]
Multispecies biofilm control	EPA, DHA	Matrix disruption; ↓ QS/virulence	Oral pathogens [76,91]

**Table 6:** Comparative overview of fatty acids, nanoparticles, polyphenols and peptides as antibiofilm agents.

Feature	Fatty Acids	Nanoparticles	Polyphenols	Peptides
Origin	Natural sources; inexpensive and non-toxic. Unsaturated fatty acids are reconsidered as antimicrobial agents due to antibiotic resistance [6].	Engineered/biofabricated; micro- and nanotechnologies are utilised to combat biofilms. Can encapsulate and deliver antimicrobial agents [170,171,172,173,174,175], including natural compounds such as carvacrol [170] or even fatty acids themselves (e.g., fatty acid-capped silver nanoparticles) [171].	Polyphenols: Natural secondary metabolites produced by plants for numerous functions, including antimicrobial defence [179]. Found abundantly in fruits, vegetables, and other plant-derived foods. Carvacrol, for example, is a monoterpene phenolic compound [102].	Part of the innate immune system (e.g., host defence peptides) [181]. Natural antibiofilm agents, often short chains of amino acids [177].
Key Mechanisms of Action	- Membrane disruption: destabilises bacterial membranes, which leads to a reduction in extracellular polymeric substances [87] and direct bactericidal action [92,93,94,95,100,101,168]. - Inhibition of adhesion: Prevent bacterial adhesion [76,119]. - Virulence factor modulation: Affect virulence factors [76,119]. - QS modulation: Induce biofilm dispersal (e.g., C2DA) [89].	- Direct antimicrobial/antibiofilm activity: Counteract infectious agents and reduce biofilm formation [171,174]. - Enhanced drug delivery: Facilitate penetration and improve antibiotic therapy [174]. - Reduction in biomass and viable cells: Reduce bacterial biomass and viable cells within biofilms [170]. - Impact on motility: Affect bacterial motility, such as swarming [170]. - Modification of surface properties: Alter viscoelasticity and fluidity of phospholipid mixtures [170].	- Multitargeted: Target cell wall, lipid membrane, membrane receptors, ion channels, bacterial metabolites, and biofilm formation [176]. Carvacrol disrupts bacterial membrane integrity, which increases permeability and leads to cell lysis, possibly due to affinity with specific membrane phospholipids [102]. - QS modulation: Interfere with QS systems [177,178,180]. - Virulence factor modulation: Suppress microbial virulence factors [179]. - Enzyme inhibition: Inhibit key bacterial enzymes [180]. - Metabolic interference: Influence bacterial metabolic processes [179].	- Membrane permeabilisation: Disrupt or degrade the membrane potential of biofilm-embedded cells [185]. - Inhibition of adhesion: Prevent initial attachment and biofilm formation [183]. - QS modulation: Downregulate QS factors and interrupt bacterial cell signalling [183,185]. - Matrix disruption: Degrade the polysaccharide and biofilm matrix [185]. - Gene downregulation: Downregulate genes responsible for biofilm formation [185].
Efficacy/Advantages	- Broad-spectrum antibiofilm activity: Notably against Gram-positive bacteria, especially *S. aureus* [87,88]. - Sub-MIC activity: Inhibit biofilms at sub-MIC [87]. - Synergistic with antibiotics: Can be combined with antibiotics to decrease biofilm inhibition and eradication concentrations [89,169]. - Reduced antibiotic resistance: Help in finding alternatives to decrease persister cell formation due to antibiotic resistance; [6]. - Prevent *E. coli* persistence: Specific fatty acids can inhibit *E. coli* persistence and biofilm formation	- Novel treatment strategies address the limitations of current antibiotics in the management of biofilm-associated infections [174]. These approaches provide high drug loading efficiency, sustained or prolonged drug release, increased stability, and improved bioavailability. They enable closer interaction with bacteria and facilitate enhanced accumulation or targeting within biomasses. These methods prevent *S. aureus* from developing drug resistance [172].	- Broad-spectrum antibacterial activity: Significant against resistant and non-resistant Gram-positive bacteria [176]. - Multitargeted mechanisms: Reduce the likelihood of resistance development [180]. - Synergistic with antibiotics: Offer promising alternatives for therapeutic strategies against antibiotic resistance when combined with antibiotics [176].	- Broad-spectrum activity: Active against multidrug-resistant bacteria and biofilm formation [181,182]. - Low resistance induction: Show low propensity to induce resistance at subinhibitory concentrations [184]. - Diverse mechanisms: Act at different stages of biofilm formation and on disparate molecular targets [183]. - In vivo efficacy: Demonstrate efficacy in various in vivo biofilm infection models [181]. - Versatile targeting: Target planktonic or biofilm-embedded bacteria [184].
Considerations	- Species specificity: Antibiofilm activity can be species-specific [87]. - High MICs: While effective at sub-MICs for biofilms, many fatty acids possess high MICs for general antimicrobial activity [87]. - Length and desaturation: No obvious rule found for optimal length and desaturation for maximal activity [87].	- Biofilm complexity: Biofilm antibiotic tolerance is different from planktonic cells, which poses ongoing therapeutic challenges. - Advancements needed: Continued research and development in nano-delivery systems are crucial [172,175].	- Less focus on antibiofilm: Most studies often prioritise antibacterial effects against suspended cells rather than antibiofilm properties [179]. - Structural diversity: Leads to a wide range of antimicrobial target diversity [178,179]	Biofilm persistence: Biofilm-linked persistent infections are difficult to treat due to resident multidrug-resistant microbes [177,181]. Resistance to antibiotics: Biofilms resist clearance by multiple antibiotics [181]. - New therapies needed: Drive the search for new antibiotic therapies due to increasing multidrug resistance [182].
Examples	Linoleic acid, petroselinic acid, oleic acid, vaccenic acid, undecanoic acid, lauric acid, N-tridecanoic acid [87,88].	Hydrophobic chitosan nanoparticles, bio-fabricated fatty acid-capped silver nanoparticles, farnesol-containing nanoparticles [170,171].	Flavonoids (e.g., quercetin, curcumin, berberine), phenolic acids (e.g., gallic acid, ferulic acid), tannins, stilbenes [102,176].	Host Defence Peptides, Antimicrobial Peptides [181,183,185].

## Data Availability

No new data were created or analyzed in this study.
